# Autonomous intelligent agents for accelerated materials discovery

**DOI:** 10.1039/d0sc01101k

**Published:** 2020-07-30

**Authors:** Joseph H. Montoya, Kirsten T. Winther, Raul A. Flores, Thomas Bligaard, Jens S. Hummelshøj, Muratahan Aykol

**Affiliations:** Toyota Research Institute Los Altos CA 94022 USA murat.aykol@tri.global; SLAC National Accelerator Laboratory Menlo Park CA 94025 USA; Department of Energy Conversion and Storage, Technical University of Denmark Lyngby Denmark

## Abstract

We present an end-to-end computational system for autonomous materials discovery. The system aims for cost-effective optimization in large, high-dimensional search spaces of materials by adopting a sequential, agent-based approach to deciding which experiments to carry out. In choosing next experiments, agents can make use of past knowledge, surrogate models, logic, thermodynamic or other physical constructs, heuristic rules, and different exploration–exploitation strategies. We show a series of examples for (i) how the discovery campaigns for finding materials satisfying a relative stability objective can be simulated to design new agents, and (ii) how those agents can be deployed in real discovery campaigns to control experiments run externally, such as the cloud-based density functional theory simulations in this work. In a sample set of 16 campaigns covering a range of binary and ternary chemistries including metal oxides, phosphides, sulfides and alloys, this autonomous platform found 383 new stable or nearly stable materials with no intervention by the researchers.

## Abstract 2

We present an end-to-end computational system for autonomous materials discovery.

## Graphical abstract

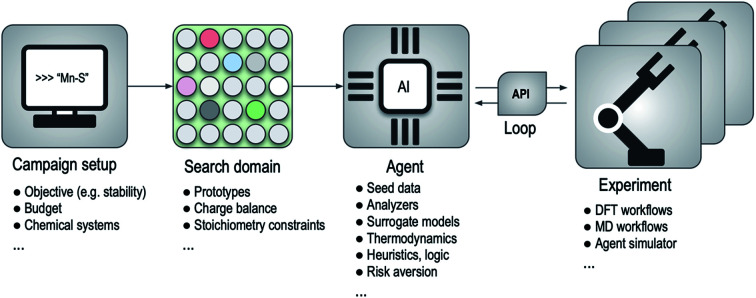

## 1. Introduction

Scientific discovery has been synonymous with serendipity, and the desire to streamline it is not new. Principles governing autonomy of agents, search of hypothesis spaces, knowledge, sequential improvement, and statistical models in scientific discovery systems had already been articulated more than two decades ago.^1–8^ For materials, autonomous discovery is being fueled by two concurrent developments: the need for new technological materials and the progress in artificial intelligence (AI).^9–11^ Recent advances in rapid generation of materials data^12–17^ have created a new frontier where computational and experimental materials research is intersecting with AI.^9,10^ This new wave of AI in materials has a theme of acceleration, enabled either by bypassing tools or methods *via* surrogate models,^18–23^ or by identification of new materials *via* adaptive schemes that combine models with decision-making approaches.^24–32^

A number of autonomous frameworks for materials discovery have been designed and demonstrated, such as optimization of carbon nanotube syntheses *via* a robotics interface;^27^ organic molecule synthesis robots^33,34^ for autonomously navigating complex chemical reaction networks with reagent-based decision-making; and composition-based autonomous search for low thermal hysteresis shape-memory alloys.^35^ Among other efforts,^36–38^ these demonstrate proof-of-concepts for autonomous discovery of materials for specific target properties or applications. In addition, open-source software packages such as ChemOS,^39,40^ which includes functionality for researcher interaction *via* natural-language-processing and robotics interfaces, and ESCALATE,^41^ which features a streamlined data capture and reporting framework, have demonstrated adaptable programming frameworks and ontologies for achieving autonomous discovery. However, there remains an opportunity for a framework which not only executes autonomous research, but facilitates the design of autonomous discovery procedures *via* a scientific method that tests the automated decision-making for their effectiveness in materials discovery.

This paper, concerned with both execution and design of autonomous discovery, introduces an end-to-end sequential framework that adopts an agent–experiment abstraction to solve complex discovery objectives in materials science. We present the framework in an application targeting discovery of stable inorganic compounds using *ab initio* calculations, a problem central to identifying new, useful candidate materials for technological applications.^42–45^ Using this framework, we show examples of (i) simulations of agents designed for stable materials discovery with existing data to gauge their performance, and (ii) active-deployment of these agents in real-world discovery campaigns of cloud-based density functional theory (DFT) calculations. We show that this autonomous platform can expand our knowledge of materials in target chemistries on user demand, and further, augment the data available in community databases like The Materials Project (MP),^13^ Open Quantum Materials Database (OQMD)^15^ or AFLOW^12^ with new, potentially useful materials. The framework is open-sourced for community development and use in other autonomous materials research settings.

## 2. Results and discussion

### 2.1 An AI framework for materials discovery

#### 2.1.1 An agent-based sequential optimization approach to materials discovery

In many cases, materials discovery can be recast as an optimization problem,^25,29,46–48^ where desired materials can be thought of as extrema of some complex objective function *f*, often to be deduced from the results of some experiments. In abstract terms, we are facing a problem that looks deceptively simple:material* = argmin *Experiment*(**material**)where material* denotes some local or global extrema of the function *Experiment*. Therefore if we can find a reasonable *f* to represent *Experiment*, a vector **X** to represent the materials, and boundaries over **X**, the expression X* = argmin *f*(**X**) could be solved with some systematic effort, hence discovery streamlined.

To be specific, let us focus on the problem of finding new inorganic materials in the most basic terms; *i.e.* previously unknown combinations of crystal structures and compositions that demonstrate a property, the most ubiquitous of which is thermodynamic stability. The effectiveness of design of experiments,^49–51^ or traditional optimization methods (*e.g.* gradient-based methods, off-the-shelf black-box or Bayesian optimizers, *etc.*) vanish quickly in such scenarios for a few reasons and we will invoke some of the early principles of discovery systems to suggest practical solutions for each. First, the experiments are often costly, and researchers have finite time and resources, such as a limited budget for running a set of experiments. The need for frugality can be satisfied by a sequential design that factors past attempts in informing its next stages. Nevertheless, rather than a fully sequential optimization framework where a single sample is run at a time, adopting a batch mode where multiple samples are run in parallel in each iteration is often desirable to minimize the total time-cost in applications where experiments take a long time.^52,53^ The advances in high-throughput computational and experimental workflows make batch mode further appealing in optimization of materials, offering a higher volume of knowledge per experiment duration, as well as potential failure mitigation by diversification of experimental parameters. Using batch mode, however, leads to some degree of loss in efficiency in using new information in decision-making compared to running a single experiment at a time, and introduces other challenges in ensuring efficient use of the allocated budget, *e.g.* how to choose the batch in each iteration for maximum return.^52,53^ Many practical decisions need to be automated to reduce this material optimization process to practice, as we show later.

Second, the *Experiment* can be highly complex, sometimes broadly thought as a “black-box”, with no gradients to aid in optimization. This complexity may necessitate the use of an array of methods to compose *f*, including not only surrogate models, but also physical or empirical models, physical laws (*e.g.* thermodynamics), or heuristics garnered by the scientists. In addition, one may need to try many such “compositions” of *f* to find the most suitable one for the task. Hence, drawing inspiration from the basic intelligent agents of AI,^54^ we adopt a similar abstraction hereafter referred to as a research agent (or simply agent), which, in our case, is a computational entity that manages the decision-making phase of the scientific-method; *i.e.* chooses candidate materials as hypotheses to test next, making use of any past or recent results and any available model, data, intuition, heuristics, logic or uncertainty as well as exploration–exploitation strategies. The agent is capable of performing this duty recursively, each time with added knowledge of recent experiments. Agents can communicate with the outside-world, namely, with external experimental facilities, by means of an experiment-specific application programming interface (API).

**Fig. 1:**
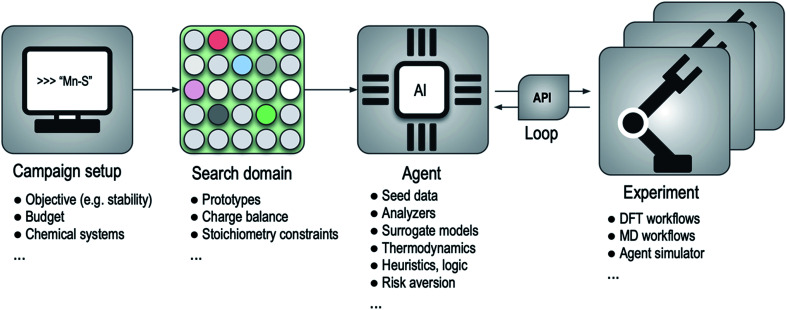
High-level flow-diagram for the agent-based sequential framework for computational autonomy for materials discovery (CAMD). Bullet points below each major component list examples or parts of the respective component. API, DFT and MD denote application programming interface, density functional theory and molecular dynamics, respectively.

An outline of this framework for computational autonomy for materials discovery (CAMD) is illustrated in Fig. 1, where the main process in a campaign is a back and forth between an agent and an experiment. Since the API translates the requests of the agent to the experimental facility and delivers the results back to the system when experiments finish, the agent is disentangled from where or how the experiments are run. This abstraction yields a modularity that allows seamless transition between experimental resources or between agents. For instance, as we show in Section 2.2, the framework can simulate the performance of a series of agents using existing data along with an “after-the-fact” experiment API that emulates running experiments but simply returns results from a lookup table, and can later swap in the relevant experiment API to run the actual experiments with an agent of choice.

In designing agents for materials discovery, a practical challenge stems from the composition–structure space of materials being infinite, and the difficulty in formulating it while ensuring distinct materials to have distinct representation vectors. Despite recent promising efforts,^55,56^ the difficulty in inversion of such vectors to materials is a compounding factor in the overall problem. A means of circumventing these issues is creating a finite search space; a domain of candidates, such as from decoration of prototype crystal structures in our current effort for finding materials of relative stability. While not restricted to it, we isolate the domain creation process from the agents (Fig. 1), as an independent stage, again to benefit from the modularity. We note also that the nature of given materials domain, particularly in the distinction between categorical and continuous variables, may strongly influence the effectiveness of a given strategy for exploring it.^57^ In our primary example, domain generation blends heuristics, such as an integral grid-based stoichiometric formula generation and chemically-selective enforcement of nominal charge-balance constraints over formulas, and prototype extraction from structure databases (see the Methods section for details).

#### 2.1.2 Specialized agents and campaigns for finding new stable materials

As outlined in Fig. 1, a “research agent” here is an abstract decision-making entity in execution of the scientific-method, and has no predisposed algorithmic form other than explicit data input and output contracts with the rest of the CAMD framework. This abstraction enables a modular software framework where designing, testing and deployment of various approaches to materials discovery become standardized and efficient. In our current implementation, all agents inherit from a hypothesis agent abstraction, which simply defines the mandatory data input to an agent to consist of two datasets; one for the seed (*i.e.* existing, known data), and one for the candidate space (*i.e.* search domain), and requiring the agent to return a subset from the candidate space that agent “hypothesizes” to meet its goal. Agents obeying this contract can be flexibly composed by the researcher and used within the CAMD framework (we refer the interested readers to our open software package outlined in Methods for further details). The crux of an agent's scientific approach therefore lies within its problem-specific design. In this work, we choose to focus on computational discovery of stable or nearly stable inorganic materials, because as a powerful indicator of realizability,^58–60^ relative stability is a general prerequisite for computer-designed materials to be useful. Hence we need to design agents for this particular purpose.

We consider several strategies in our agent-design for stable material discovery. As a common design element, agents are allowed to take existing results from DFT calculations that were already run, train formation energy regression models, modify predictions on the candidate space having the objective of stability in mind (*e.g.* by tagging on an uncertainty derived component or other exploration–exploitation strategies), construct a thermodynamic energy-composition convex-hull and use distance to this estimated hull as part of their decisions. The use of convex-hull within agents is a concrete example of a physical construct that is leveraged in the form of relational-information between materials, that is otherwise not easy to incorporate as part of standard optimization frameworks. Agents can also use logic; *e.g.*, current agents enforce a floor for how low predicted energies can go, so they do not inadvertently skew the results in early iterations where energy predictions might be poor. Or they can use heuristics, *e.g.* to minimize the risk of acquiring similar points, or terminate if their performance is not satisfactory. Further details of agents are provided in the Methods section.

There are a large array of approaches for how acquisitions can be decided for finding materials that meet the objective of stability, often taking into account the variance in model predictions, such as the probability of improvement, expected improvement, lower (or upper) confidence bound or greedy (including *ε*-greedy) approaches.^25,61,62^ We frequently use the latter two, as they more easily generalize to optimization in the data-regimes we navigate. Some related algorithms the current set of agents adopt include query-by-committee (QBC)^63,64^ which can decorate a learner with variance in predictions, ensemble learners where prediction variances can be approximated in a similar way, or Bayesian approaches, including Gaussian process (GP) and its variants which circumvent its unfavorable computational complexity.^65^ Voronoi-based descriptors by Ward *et al.*^66^ are incorporated to represent materials in models, and for stability agents that require a regressor, we use a simple two hidden-layer (84 × 50) fully-connected neural network (NN) (see Methods). We found that for the present application, these choices for the formation energy models provide a good compromise in terms of accuracy and the speed of model training and prediction.

The value delivered by the discovery system should be measured carefully against baselines. A randomly choosing (RC) agent is therefore always simulated for comparison. For the task of finding new materials, however, this agent is not too difficult to outperform, unlike an active-learning scenario, where the goal is to minimize the variance over the prediction. In that case, an RC is a strong baseline. Thus, for discovery tasks, we simulate additional “one-shot” baselines, to test whether the sequential learning paradigm (*i.e.* incremental improvement of agent) is helping the discovery or not. These, unlike an RC agent, are difficult to beat, as they harness models trained on the entire past data, and can use acquisition techniques, but in contrast to sequential agents with access to accumulating data, are simply not given the opportunity to learn from each prior iteration. They make their choices in a chemical system, in one-shot, at the same overall budget as other simulated agents.

### 2.2 Campaign simulations with agents for materials discovery

#### 2.2.1 Overall structure of campaign simulations for agent design

We designed an array of agents that aim for discovering (nearly) stable materials and tested their performance in simulations in various binary chemical spaces using the data available in the OQMD.^14,15^ Since these agents are after new stable materials, we use the unique Inorganic Crystal Structure Database (ICSD)^67^ derived entries in OQMD with about ∼36 thousand entries as the standard seed data for all agents. Hence, during agent design *via* simulations, the agents exclusively choose from hypothetical (prototype-derived) OQMD entries (unseen to the agent), set aside upfront as the “candidate space” of crystal structures (see Methods). The goal of the agents is to find the stable ones among these hypothetical candidates. This design ensures the agent simulations closely mimic the actual discovery campaigns we want to deploy later (Section 2.3). We give the simulated agents a budget of 50 experiment requests in each round. In the particular case of these after-the-fact simulations, the system uses an experiment emulator that reads the results from a table and communicates immediately back to the agent as if DFT simulations were run. For these agent simulations, we first select the Fe–X binary systems, where X is any element other than Fe, as they provide a large enough candidate set of 1628 entries, all hypothetical, and span a range of material classes: from metallic to covalent. Among these candidates, only about 220 are within 0.1 eV per atom of the convex-hull (hereafter we refer to these as “stable” for simplicity) when measured against the cumulative set containing the ICSD-derived entries as well as the hypothetical entries themselves.

#### 2.2.2 Baselines and simple agents: decisions with minimal complexity

**Fig. 2:**
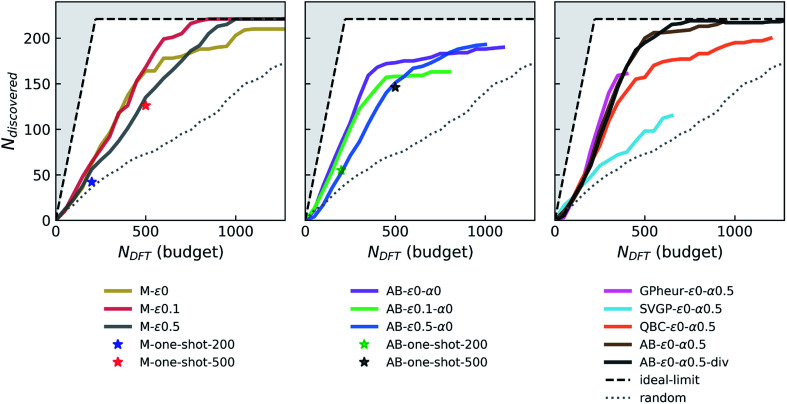
Simulated performance of various agents in discovering stable Fe–X binary compounds. *N*_discovered_ and *N*_DFT_ correspond to the total number of materials that are within 0.1 eV per atom of the instantaneous convex-hull, and the DFT budget (in terms of total number of DFT calculations agent is allowed to request), respectively. Agents are labeled as follows: M, AB, GPheur, SVGP and QBC which respectively correspond to “greedy” agent with a surrogate model, AdaBoost based agent, heuristic GP agent, stochastic variational GP agent, and query-by-committee based agent. The parameters *ε* (fraction of random exploration) and *α* (mixing parameter for uncertainty) are followed by their respective values in labels. The “one-shot” points refer to direct acquisition of *N* = 200 or 500 candidates, with the noted agent (M refers to the NN regressor, and AB refers to the same regressor boosted with AdaBoost), with no sequential (batch-mode) acquisitions. Presence of “div” in the label means the agent uses diversification. Ideal-limit shows the maximum achievable performance, *i.e.* if every agent-designated material was deemed stable and therefore “discovered”, outside of which is not accessible by any agent and hence grayed out.

Simulated performance of different agents are displayed in Fig. 2. We start with simple designs and incrementally build complexity into the agents. The simplest of all; randomly-choosing agent takes the entire candidate set to discover all stable candidates. The next class are the “greedy” agents (marked as M in Fig. 2), which pick those candidates projected to be within the stability threshold (and ranked on the basis of their distance to hull). In each iteration, these agents train a formation energy model (a NN described above) on the seed data (combination of the initial seed and results of its experiments) and make predictions on the remaining candidate set, followed by an aggregated convex-hull computation for all candidates and existing data to measure their distances to the hull. We find that this relatively simple greedy agent (M-*ε*_0_) yields a discovery rate (*i.e.* slope) considerably better than the random agent. We also find that it outperforms its “one-shot” analogues, demonstrating that the sequential strategy is indeed beneficial. After finding about ⅔ of the stable materials (at about ∼500 DFT requests), this agent enters a regime of mild “saturation”, and its performance drops. As we will show later, a simple explanation of saturation is that the accuracy of the energy model is not sufficiently high to distinguish the remaining stable materials from the rest of the candidate pool at later stages of the campaign. This causes the greedy-agent to get “stuck”. A standard way to mitigate this problem is letting the agent not only exploit its best predictions, but also explore some candidates randomly, by a fraction *ε* of its budget. We observe that such an *ε*-greedy agent with *ε* of 0.1 discovers at the same rate as its fully-greedy counterpart, but does not show any notable saturation until the candidate set is exhausted. As *ε* gets larger (*e.g. ε* = 0.5), however, the discovery rate starts to decrease.

#### 2.2.3 Role of energy prediction accuracy in agent performance

The next question we ask is how much role the underlying energy regressor is playing in the performance of the agent. The cross-validation (CV) mean absolute error (MAE) of the original NN we use remains close to 95 meV per atom (with our seed of ∼36 000 ICSD-derived OQMD entries and newly “acquired” examples in each campaign), similar to the random forest model trained on ∼30 000 examples in the original study that introduced the Voronoi-based descriptors we use.^66^ We did not see a notable drift from this performance with growing data set size or switching between models with enough complexity (*e.g.* random forest *vs.* NN). However, we find that adaptive boosting (AdaBoost, or simply AB) of the original regressor notably increases the model accuracy, reducing the CV-MAE with the same data to about 77 meV per atom. This boosting yields an improvement in the discovery rate (Fig. 2, second panel) for the greedy agent (AB-*ε*_0_-*α*_0_), but a pronounced saturation happens at about the same level as the non-boosted greedy agent. Interestingly, switching to an *ε*-greedy strategy for the boosted agent (*e.g.* AB-*ε*_0.1_-*α*_0_) does not help avoid early saturation (which will be studied in detail in a later section). Since results of the DFT or convex-hull computations do not have any notable uncertainty element, the accuracy of the formation energy model remains as the main bottleneck in the overall performance of the agents. Considerations of prediction uncertainties in decisions could therefore be useful for improving the agent performance.

#### 2.2.4 Agents that incorporate uncertainties in decisions

As a general approach, we adopt lower confidence bound; LCB (often studied as upper confidence bound depending on the context),^61^ a well-known strategy that reflects “optimism in the face of uncertainty” to address exploration–exploitation threshold. In the current LCB approach, formation energy predictions, Δ*Ĥ*_*f*_ are combined with their uncertainty as LCB(**X**) = Δ*Ĥ*_*f*_ − *ασ*(**X**) where *α* is a positive mixing parameter for uncertainty *σ*, and the rest of the pipeline is similar to the greedy agent. Thus, we need formation energy models that can decorate their predictions with uncertainties, and below we explore several such options.

While a GP is the “go-to” Bayesian approach for this purpose, its computational complexity prohibits its straightforward use in the data regimes (>30 000 examples with 273 dimensional representations) we operate in. We first try a simple workaround: a GP is trained on a seed that combines ICSD-derived OQMD compounds in Fe–X chemistries, with 5000 additional samples randomly drawn from the rest of the ICSD-OQMD seed. We find that an agent employing this heuristic GP strategy (GPheur-*ε*_0_-*α*_0.5_) is on par with the boosted greedy agents, despite its lower MAE of ∼110 meV per atom, but saturates early like the fully-greedy agents. Despite its ability to handle big data in a GP setting, stochastic variational inference GP^65^ yielded relatively large MAEs (∼130 to 140 meV per atom) with the surrogate matrix sizes we could afford, and using the same LCB strategy, the agent SVGP-*ε*_0_-*α*_0.5_ displayed a relatively poor performance (third panel in Fig. 2).

Given the above difficulties, we turn to decorating predictions of non-Bayesian regression models with uncertainty estimates. QBC is an ensemble method analogous to bagging and estimates prediction variance from the disagreement of the committee members (each trained on a subset of data), and can be used for this purpose.^64^ A QBC agent that uses LCB (*α* = 0.5) with our standard NN regressor did not produce results better than a fully-greedy agent. The random acquisitions, as in *ε*-greedy agent, seem to play a more significant role than uncertainty estimates from QBC, to overcome the observed saturation. Given that boosting forms an ensemble of models, we can decorate predictions of AB with proxies for uncertainties derived from the variance among that of each regressor in the ensemble. An AB agent that uses such uncertainties with LCB and *α* = 0.5 (AB-*ε*_0_-*α*_0.5_ in Fig. 2) has the same discovery rate as its boost-only parents, but outperforms them by saturating much later, at a point where about only 20 stable materials left in the candidate pool. This is so far the best performing agent.

#### 2.2.5 Transferability of agents

**Fig. 3:**
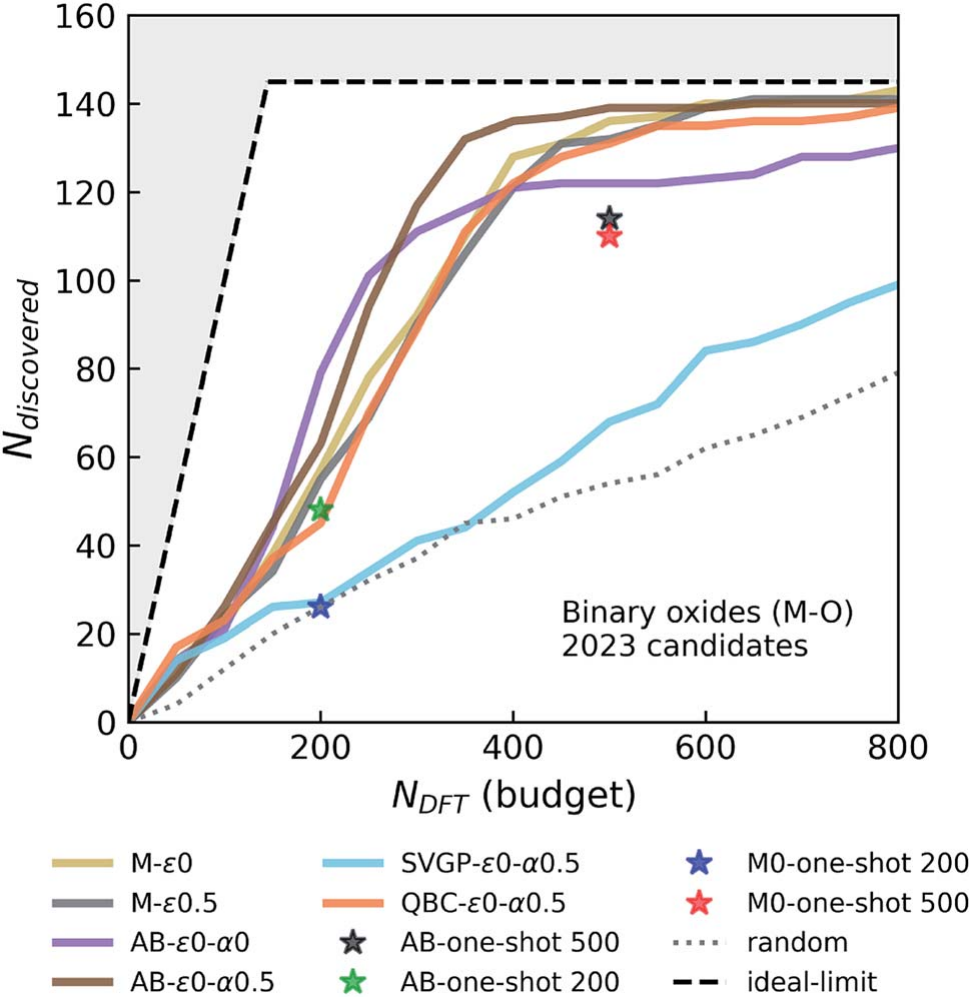
Simulated performance of various agents in discovering stable M–O binary compounds. *N*_discovered_ and *N*_DFT_ correspond to the total number of materials that are within 0.1 eV per atom of the instantaneous convex-hull, and the DFT budget (in terms of total number of DFT calculations agent is allowed to request), respectively. See the caption of Fig. 2 for the descriptions of the layout and agent labels.

The performance of the agents evaluated by simulations in the Fe–X dataset is not guaranteed on all datasets, but since this dataset has broad structural and compositional diversity with 83 unique elements considered in position of X, we expect it to be fairly representative of the relative performances of agents in other chemistries. To further test if this statement holds, we design an alternative candidate set of M–O binaries chemically orthogonal to the previous one, by following a similar approach; *i.e.* setting aside all hypothetical M–O binaries in the OQMD (which contains 84 such unique binary systems) as the candidate set, and keeping the remaining OQMD entries with ICSD-origin (including the ICSD based M–O as well) as our seed data, to form a strong baseline for agents to compete against (as was done for Fe–X). This candidate space has a size of 2023 unique entries, 145 of which are within 0.1 eV per atom of the convex-hull. The performance of primary agents selected from Fig. 2 in this particular scenario are displayed in Fig. 3. We find that the performance trends from Fe–X campaigns reasonably translate to the M–O campaigns; *e.g.* all performant agents still outperform the random baseline by a substantial margin, and performance of AB agents are higher than M agents, and the greedy AB-*ε*_0_-*α*_0_ saturates early, which is again overcome by the LCB strategy in AB-*ε*_0_-*α*_0.5_. With this example, we are able to confirm that the observed relative performances of agents in the broad Fe–X chemistries are fairly transferable to others, such as M–O, which has almost no overlap with the former (except for of course the Fe–O system).

#### 2.2.6 Variability in agent performance due to incomplete phase information

Campaigns described so far used a fixed seed encompassing the unique ICSD based entries in the OQMD excluding only the hypothetical phases in target binary chemistries (to use them as candidates). This fixed seed scenario provides a strong baseline for agents to perform against, and mimics the actual deployment scenario in active campaigns closely (described in the Section 2.3). However, this deterministic scenario does not reflect the potential variability in performance that agents may experience due to partial, incomplete phase information in seed data in target chemistries. Since completeness of phase information is difficult, if not impossible, to fully ensure (not only in under-explored systems but even in thoroughly-studied systems, where there may still be materials awaiting discovery), we would like to understand its influence on the performance of the agents by designing relevant campaigns that are as close as possible to the deterministic ones described before.

**Fig. 4:**
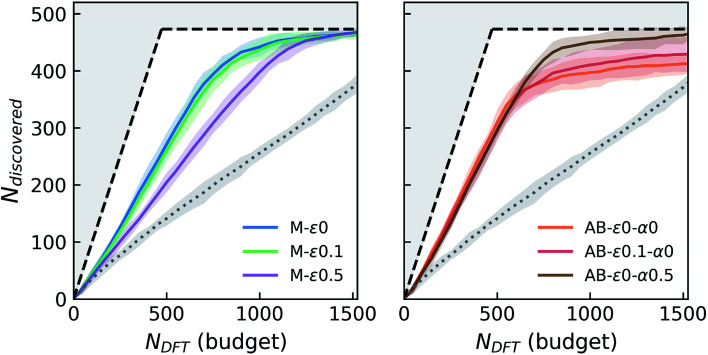
Simulated performance of selected agents under variable-seed conditions. For each agent, the solid line and the shaded area around it show the average number of discoveries and the ±2*σ* (95%) confidence interval around these averages, from at least eight random campaign initializations. See the caption of Fig. 2 for the descriptions of the layout and agent labels.

In these variable-seed campaigns, we still focus on the Fe–X chemistry due to its chemical diversity and candidate set size, as well as for consistency with the previous campaigns, but this time we remove all Fe–X phases from the seed regardless of its origin, and reserve all of the 1933 Fe–X phases as candidates. We start these campaigns by randomly choosing and adding 50 candidates from this Fe–X candidate space to the above-mentioned seed. While we keep the chemistry and other settings (*e.g.* number of queries in each iteration) consistent as much as possible with Fig. 2, these new campaigns by design have a slightly larger candidate pool, and hence more stable materials (473 within 0.1 eV per atom of the convex-hull) available compared to prior deterministic campaigns. For each agent, we repeatedly run these campaign simulations with varying initial seeds at least eight times for the primary agents of interests that we selected from Fig. 2. The results of these variable-seed campaigns are presented in Fig. 4, where ±2*σ* confidence bounds are shown around the mean performance at each iteration of the campaign. Although there are minor performance differences compared to Fig. 2 due above-mentioned slight differences in campaign structure (for example M-*ε*_0_ and M-*ε*_0.1_ are closer now, but variation in performance with *ε* is still present as seen for M-*ε*_0.5_), most notable aspects, such as the improvements in performance with AB agents, their early saturation in greedy or *ε*-greedy strategies, and further improvement with LCB in AB, are all observed in this setting as well. Fig. 4 clearly shows that the confidence intervals are relatively tight, especially in the early stages of the campaigns, and tend to broaden only slightly as campaigns progress, the largest *σ* remaining within ∼13 compounds in AB agents. These results indicate that the incomplete phase information in partially explored chemical spaces do not result in significant variations in agent performance.

#### 2.2.7 A closer inspection of the decision-making in agents

**Fig. 5:**
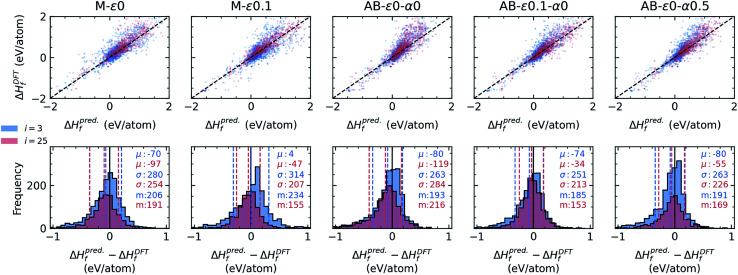
Analysis of the errors in predicted energies of candidates by various energy-models underlying several agents at early (iteration = 3) and later (iteration = 25) stages of respective campaigns. Top panels show parity plots comparing predicted formation energies to DFT values for candidates at the selected early and late stages of each campaign, where dashed-lines are a guide for the eye depicting ideal fit (*i.e. x* = *y*). The bottom panels show corresponding histograms of the difference between predicted and DFT values of formation energies for candidates in the same stages of the campaign as the top panels. Each same-colored set of vertical dashed-lines correspond from left to right to *μ* − *σ*, *μ* and *μ* + *σ* where *μ* and *σ* are the mean and standard deviation of their corresponding (same colored) distributions at two separate stages shown. Corresponding *μ* and *σ* (in units of meV per atom for clarity) are also shown as insets along with MAE (as “m”), color-coded the same way. The agent labels are shown above the parity plots.

While our focus thus far has been designing and simulating a variety of agents for stable materials discovery to identify the ones that are the most performant, the mechanisms behind the observed performance evolution trends in campaigns, such as the counter-intuitive early saturation of certain AB agents in Fig. 2–4, are important to understand to design better agents in the future. As discussed before, the primary source of error in the current sequential learning application is the formation energy models trained and used by the agents. Therefore, in Fig. 5, we inspect the prediction errors in formation energies in the remaining candidate set during two distinct stages of each campaign (consistently selected to reflect earlier and later stages – see the figure caption) by the specific regression models for the primary agents discussed before. We immediately observe that prediction errors in remaining candidates are close to normal distributions. However, AB-*ε*_0_-*α*_0_ (*i.e.* the fully greedy AB agent), despite its better CV-MAE of ∼77 meV per atom in seed data, shows a larger bias towards lower formation energies in its predictions, accompanied by a visible skew in the error distribution and parity plots, which persist from earlier to later stages of the campaign. With no explicit acquisition mechanisms to mitigate this issue, AB-*ε*_0_-*α*_0_ keeps acquiring points where it is fairly accurate (as evident from the evolution of its error distribution). In turn, this agent's accuracy does not improve for candidates near lower and upper tails of its error distribution (in fact its overall MAE in candidate set deteriorates as campaign evolves), and once it mostly exhausts the candidates from the accurate (middle) region of the same distribution, AB-*ε*_0_-*α*_0_ gets stuck (*i.e.* saturates early). Its simpler greedy version, M-*ε*_0_, which also lacks any explicit mechanism to mitigate such issues, does not exhibit the same degree of early saturation in Fig. 2–4 (only a mild tendency in Fig. 2). This observation indicates that with less-biased predictions (and higher normality of its error distribution) seen in Fig. 5, M-*ε*_0_'s acquisitions tend to more evenly sample the candidates from the broader error range, opposite to AB-*ε*_0_-*α*_0_, in turn not leading to such pronounced saturation. In other words, regardless of whether the agent has better CV-MAE in seed data, biased and/or skewed predictions in candidate space seem to be a primary cause of early saturation. The finite *ε* helps AB-*ε*_0.1_-*α*_0_ more evenly sample the candidate space in the entire error distribution. While it was not clear from the deterministic campaigns in Fig. 2, we observe in variable-seed campaigns in Fig. 4 that the finite *ε* indeed helps AB-*ε*_0.1_-*α*_0_ improve its saturation point on average, not substantially but at least to a statistically measurable degree. In AB-*ε*_0_-*α*_0.5_, not only the underlying model's accuracy improves, but the LCB strategy that explicitly considers the uncertainty of model's predictions in candidate space turns out to be more effective in identifying the remaining stable candidates as campaign advances.

#### 2.2.8 Summary of agent simulations and transitioning to active deployment

The takeaway from stable material agent simulations is that the saturation point of agents can be enhanced by adopting different exploration–exploitation tradeoff strategies, namely, *ε*-greedy strategies or incorporating uncertainty *via* lower confidence bound. The discovery rate can be improved, to some extent, by using more accurate models for formation energy. Any significant improvement is unlikely to be achieved by further tuning of the parameters of the presented strategies. Prior to the deployment of the best performing agent in active campaigns, we augment it with a risk-aversion strategy that diversifies the acquisitions (AB-*ε*_0_-*α*_0.5_-div in Fig. 2). As expected, when simulated with OQMD data (which already went through structural uniqueness filters), this strategy does not affect the performance, but is put in place to ensure that in prototype-based searching in active campaigns, where similar structures are more likely to be encountered, the agent can prioritize acquiring dissimilar ones (see the Methods section).

### 2.3 Active deployment of agents: DFT-based campaigns of materials discovery

#### 2.3.1 Overall structure of active DFT-based campaigns for stable materials discovery

In this section, we demonstrate the deployment of the best agent designed in the previous section in active DFT-based stable material discovery campaigns in a diverse set of sample chemistries. The described abstraction of *Agents* and *Experiments* enables a seamless transition from simulations of campaigns to production deployment of a materials discovery workflow. As outlined in Fig. 1, the only additional tasks we need in active deployment are: (i) to create the domain of inputs which may be tested in experiments (*i.e.* the candidate space), and (ii) the experiment which will acquire new data and augment the seed data with which the campaign begins and continues.

For the domain creation, we use an approach that decorates crystal structure prototypes derived from databases of existing materials, combined with chemical, stoichiometric and charge-balance heuristics (see the Methods section). Briefly, the inputs to a stable materials discovery campaign are a list of elements which define the chemical system in which materials are to be discovered. With these elements, reasonable chemical formulas are generated algorithmically using heuristics from charge balance and permissible integral stoichiometric coefficients of elements in formulas. Candidate structures at these compositions are generated from the large array of anonymized (prototype) crystal structures. This process of domain generation renders a dataframe of structures keyed by element-specific stoichiometry and structure prototype and provides it to the campaign as the “candidate data”. The “seed data” is provided as the ICSD-derived entries in the OQMD (as explained before), because they reasonably capture the current stability landscape from experimentally known materials, and hence form a strong baseline against which to measure the stability of new findings. In effect, the energy-composition convex-hull construction for determination of the stability of new discoveries explicitly includes experimental data to the extent possible in the present framework, ensuring the predictions are valid in comparison to real, known materials. We note that these are merely the implementation defaults, and framework allows using other choices for all these components.

Defining experiments typically requires more effort than defining the campaign domain, as ensuring compatibility of the experimental data generated and the existing seed data may require knowledge of that seed data's provenance. For our initial implementation, we employ a DFT workflow that mirrors calculations done by the OQMD (see the Methods section), which ensures compatibility with DFT data that already exists therein. We employ a strict time limit for DFT runs to prioritize rapid turnover, and therefore agents may often receive a fraction of their requests back (further explained in the Methods section). Upon completion of a given experiment, a stability analyzer computes the convex hull corresponding to data from both new experimental results and prior seed data. We note that stability is an aggregate property of the dataset and thus requires a post-processing step independent of the experiment that includes data from the same seed data used as part of the agent. This batch-mode sequential workflow of the agent and the experiment iterating back and forth continues until the budget is exhausted or the campaign is terminated for one of the reasons outlined in Methods. A post-campaign structure analysis is performed, as prototype derived structures can often relax into a similar structure after the DFT relaxation, and hence become duplicates. The settings and other practical details of active campaigns reported here are listed in the Methods section.

#### 2.3.2 Active discovery campaigns in diverse chemistries

**Fig. 6:**
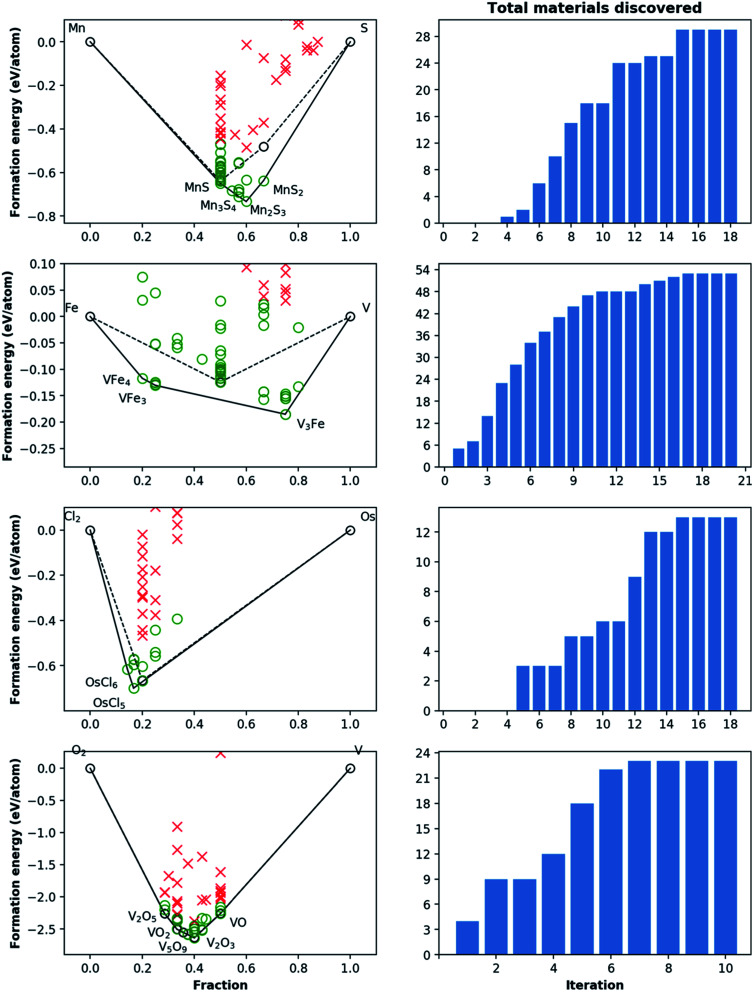
Phase diagram and cumulative discovery histograms for CAMD campaigns in Mn–S, Fe–V, Os–Cl, and V–O chemical systems. The 2-D phase diagrams are shown on the left with both pre-campaign convex hull (dashed line) and post-campaign convex hull (solid line). Material discoveries, *i.e.* computed structures within 0.2 eV per atom of the convex hull, are shown as green circles, while materials higher than this threshold are shown as red crosses. The number of total materials discovered for each chemical system as the campaign iterations progress are shown in the right-hand bar charts. For materials from pre-campaign convex-hulls (*i.e.* existing materials), only the most stable ones are shown for simplicity.

**Fig. 7:**
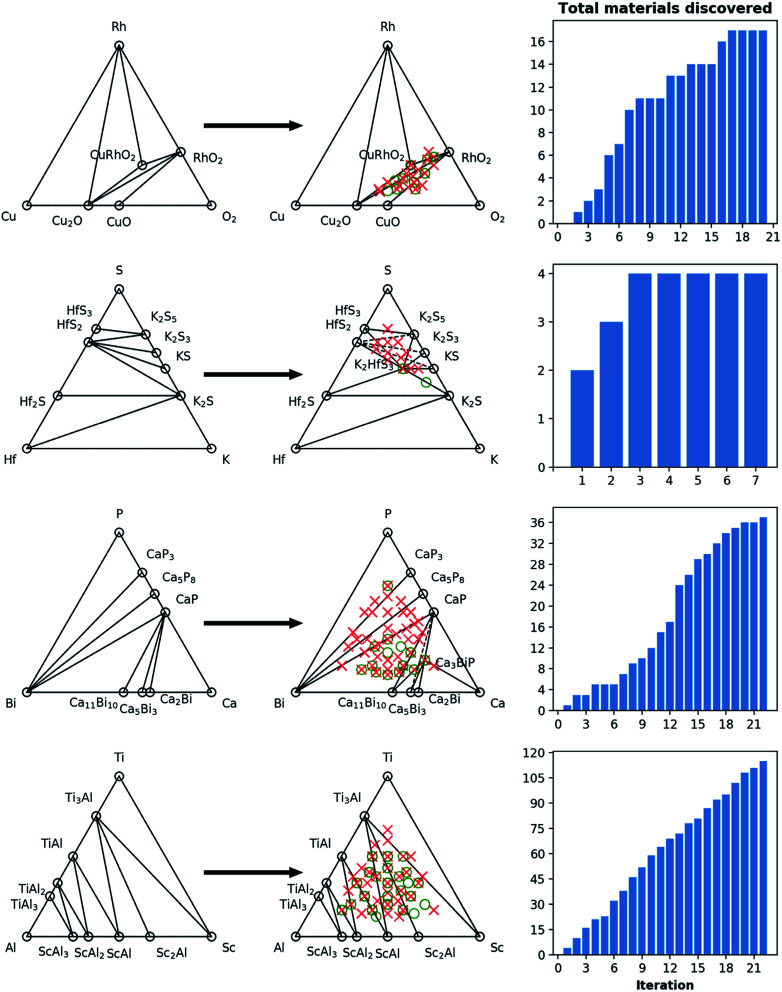
Phase diagram and cumulative discovery histograms for CAMD campaigns in Cu–Rh–O, Hf–K–S, Ca–Bi–P, and Al–Ti–Sc chemical systems. The ternary phase diagrams are shown on the left with both pre-campaign convex hull (left column) and post-campaign convex hull (center column). Material discoveries, *i.e.* computed structures within 0.2 eV per atom of the convex hull, are shown as green circles, while materials higher than this threshold are shown as red circles. The number of total materials discovered for each chemical system as the campaign iterations progress are shown in the right-hand bar charts. Note that, for Hf–K–S and Ca–Bi–P, discoveries K_2_HfS_3_ and Ca_3_BiP are on the convex-hull, *i.e.* current ground-states at those compositions.

To demonstrate the active deployment of agents for materials discovery campaigns, we present results from a diverse set of chemical systems such as Mn–S, Fe–V, Os–Cl, V–O, Cu–Rh–O, Hf–K–S, Ca–Bi–P, and Al–Ti–Sc in Fig. 6 and 7, and statistics pertaining to all 16 campaigns run in this work are provided in Table 1. These examples are picked to cover a range of material classes (*e.g.* oxides, sulfides, phosphides and alloys) or bonding types (*e.g.* metallic, covalent and ionic), as well as different degrees of prior experimental exploration in the literature, and different degrees of success in discovery by the agents. Many of these systems are relatively common, well-explored chemistries populated with experimentally known compounds, and hence serve as stringent tests for the framework. The evolution of the convex hulls for binary and ternary systems can be clearly seen in Fig. 6 and 7, where the pre-campaign convex-hulls show material structures in the OQMD that have an experimental source.

**Table 1:** Campaign statistics for the chemical systems studied in this work. Gross discovery represents the total number of new materials within 0.2 eV per atom of the convex-hull at the end of the campaign, prior to post-campaign structure analysis. Duplicate discovery column lists how many of the structures among the gross discoveries were found to be duplicates of others. We further checked how many of the discovered structures relaxed into ICSD-derived entries in OQMD, listed as experimental in the table. Final column lists the total number (383) of explicitly unique, non-experimental structures

System	Agent requests	DFT received	Gross discovery	Duplicate discovery	Experimental	Unique discovery
B–Fe	152	146	32	28	3	**19**
C–Fe	91	81	37	14	2	**26**
Cl–Os	101	70	13	1	1	**11**
Fe–N	66	47	17	10	1	**10**
Fe–S	158	133	22	23	6	**14**
Fe–V	147	130	53	30	1	**32**
Mn–S	99	73	29	5	2	**22**
Ni–S	220	191	94	30	6	**66**
O–V	66	52	23	5	3	**17**
S–V	174	129	31	22	3	**20**
Al–Sc–Ti	216	199	115	32	0	**90**
Bi–Ca–P	220	157	37	10	0	**27**
Ca–Mo–P	73	53	6	2	0	**4**
Cu–O–Rh	123	72	17	4	1	**14**
Hf–K–P	89	63	8	4	0	**7**
Hf–K–S	38	23	4	0	0	**4**

**Fig. 8:**
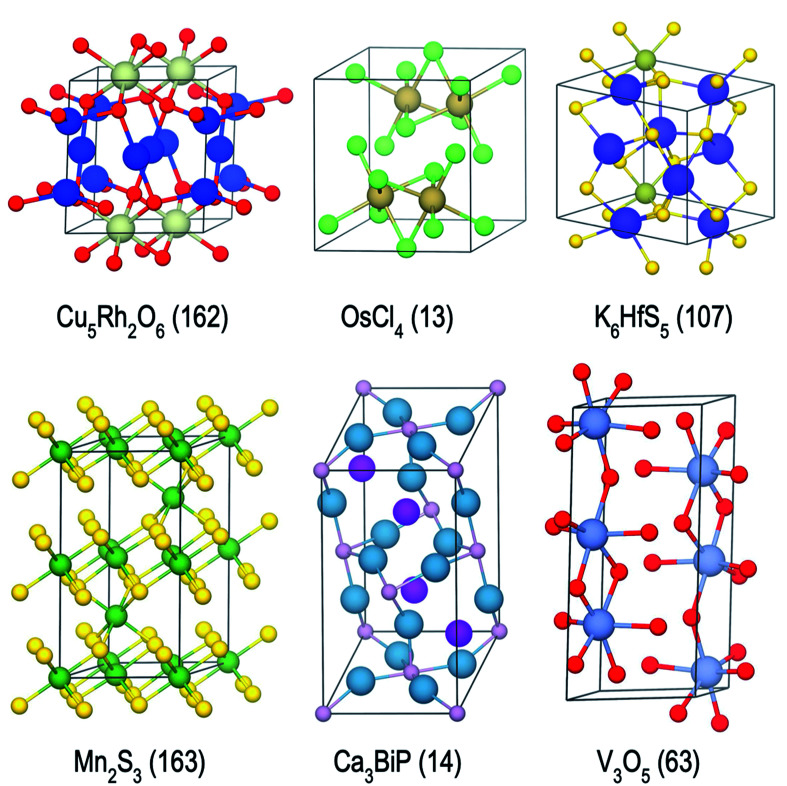
Examples of stable or nearly stable crystal structures found in the present campaigns. Space group numbers are given in parentheses.

For the 16 chemically-diverse campaigns presented in this manuscript, we report 383 autonomously-discovered distinct structures that could not be matched to an experimental entry in the OQMD and are within 0.2 eV per atom of the convex hull. The discovery rate for unique structures within this threshold range from around 10% to 45% across different campaigns (Table 1), averaging close to 22%. Out of these structures, 13 are on the convex-hull, and 36 are within 0.025 eV per atom, 67 are within 0.05 eV per atom and 153 are within 0.1 eV per atom of the convex-hull. Of the structures discovered as unique, only 9 are found in the MP database, suggesting that 374 of the discovered structures have not been previously reported there either. In addition, we note that the structures generated by CAMD have a natural structural diversity that is limited only by the structural diversity of the seed from which the prototype set is generated. From the 383 discovered structures, 7 crystal systems and 87 distinct space groups are represented. Furthermore, it can be observed in Fig. 6 and 7 that the breadth of compositions CAMD samples varies notably across different chemistries. Particularly in spaces where a charge-balance can be enforced (*e.g.* Cu–Rh–O), agents naturally focus mostly on the respective parts of the phase diagram enclosed by existing charge-balanced compounds, avoiding potentially unphysical compositions (see Methods for details). While this choice comes at the expense of potentially missing a few plausible candidates (*e.g.* peroxides or superoxides), we find it to be essential to ensure efficient use of computational resources in the current “exploratory” campaigns. In cases when charge-balance is not as plausible and hence is not enforced, the composition space broadens (*e.g.* Ca–Bi–P and Al–Sc–Ti). Some of the stable or nearly stable structures identified by CAMD are displayed in Fig. 8.

We find it encouraging that the CAMD framework identified many new, unique stable (or nearly stable) phases in this diverse set of binary and ternary chemical systems, even in systems that one expects to have been thoroughly-explored, such as Mn–S or Fe–V. Nevertheless, a quick survey for these two examples reveals that for the Mn–S system, Okamoto^68^ mentions that the S-rich side is still speculative due to insufficient experimental data, and for the Fe–V system, Bloch *et al.*^69^ performed an *ab initio* high-throughput search and found new ordered Fe_3_V and FeV_3_ phases, and further provide experimental evidence that kinetic barriers to formation of such previously unobserved phases in this system can be overcome by hydrogen absorption. These examples hint at the need to revisit even such well-known systems for new materials, and also show CAMD can rapidly uncover such gaps in our knowledge of materials without any intervention. While searches in MP^13^ and OQMD^15^ show that both databases naturally identified several hypothetical structures in the current chemical systems (and many others) as part of high-throughput prototyping studies run over the course of years, we showed that by streamlining the discovery process, CAMD far exceeds prior work in terms of the number of viable unique material structures and the breadth of new compositions such structures are found at (Table 1). Thus, our current, and future results as they are being produced by the present framework are expected to be valuable additions to such open material databases.^12–14^

## 3. Outlook and open questions

The CAMD framework is suitable for sequential campaigns in materials space in general, but we expect it to be more effective in optimization efforts for identifying new inorganic materials as showcased in this study, where the search space is large and representations of entries are high-dimensional (*i.e.* thousands of candidates and complex vector representations), with no obvious gradients to trace, and where acquisition decisions require blending of multiple scientific paradigms (such as machine-learning, phase-diagram construction, risk-aversion algorithms, *etc.*) under strict budget constraints. The design of the framework, and in particular the agents, as well as many of the hyperparameters or practical implementation details of the system are geared towards our goal of achieving rapid searching of entire chemical systems at minimal computational cost. While there are no restrictions to investigating individual compositions, as their core advantage, the agents in this work process entire systems and are capable of taking into account the relative stability of materials not only against their polymorphs but against decomposition into other materials in their entire chemical systems.

The framework currently assumes *a priori* creation of a search domain, and hence is not generative in the sense that it would not consider an entirely new crystal structure that does not exist in our list of chemically anonymized prototypes derived from databases of known materials. But constraining the search space this way enables speed, in other words, we can scan entire chemical spaces (such as binaries or ternaries) for new stable materials at relatively small computational costs. With the current settings, a budget of maximum 220 DFT calculations yields many new stable (or nearly stable) materials in entire chemical systems (Table 1), often spending a lot less than the allocated budget. For these reasons, we expect that the present use case of finding new stable materials will be complementary to the existing approaches to crystal structure prediction.^70–72^ We should also emphasize there is no obstacle against the implementation of a generative approach in the presented framework. Such generative-agents, for example, can hypothesize the same way and suggest new structures while they are active or a generative approach can deliver a large set of candidate structures upfront to form the search domain.

We expect that the future work can extend the framework to achieve improved performance in the present application, and to add new target applications and functionality. For example, more intelligent agents can be developed for tasks including and beyond finding stable materials, such as studies that consider other material properties and specific material applications. For this purpose, both traditional^73^ and newly advanced *a priori*^74^ multi-objective approaches may serve as useful components of the CAMD discovery agents. Those agents can adapt new representations, surrogate models, rules, empirical relations, physical or chemical laws *etc.* In our initial implementation to find stable inorganic materials, no entropic effects are considered in the determination of whether a material is to be marked as a discovery. We note that these may serve an important role and are an objective for future implementations of our experimental and analysis functionality, particularly in alloy and higher-order systems.^75,76^ Since thermodynamically accessible energy ranges of making polymorphs vary across material classes,^58,59^ agents that utilize a chemically-informed hull-distance threshold are likely to deliver more performant, and less-biased campaigns across chemistries. *A priori* identification of underexplored chemical spaces with higher likelihood of discoveries can improve performance in exploratory searches.^43,77^ Incorporation of cost estimation of acquisitions, dynamic budgeting, and better error handling (*e.g.* whether to attempt to heal a failed request) are planned as additions to the framework. Agents can adopt strategies that alter their hyperparameters (*e.g. ε* and *α* discussed above) in response to observed performance metrics. Besides, it is foreseeable that, as if forming a team of scientists, a team of agents would be able to attack a scientific problem from different angles, or agents can demonstrate self-improvement skills in campaigns, beyond minimizing the variance of surrogate models and would be more risk averse. Intersection with, and potential benefits from other black-box optimization approaches and reinforcement learning are expected.^78^

## 4. Conclusion

In this work, we presented a sequential, agent-based optimization framework for complex scientific objectives, designed explicitly with the constraints and utility of materials discovery in mind. The framework is designed to be modular such that it allows simulations of research agents before their active deployment in real discovery campaigns, enabling their efficient, *a priori* design by the human researchers. We demonstrated agent simulations in a case study of Fe–X systems and showed how complexity can be built into agents, from baseline one-shot models to uncertainty-driven decision-making. We further demonstrated active DFT-based deployment of agents we designed in 16 sample chemistries. The framework found hundreds of new, structurally-unique materials in these chemistries with no human intervention, validating, and also demonstrating the utility of having an end-to-end framework for discovery of new materials autonomously. The supporting code is open-sourced for community use.

## 5. Methods

### 5.1 Code, models and data availability

We have developed an open-source python-package, named computational autonomy for materials discovery (CAMD) to carry out the work presented in this publication, available at https://github.com/TRI-AMDD/CAMD. The CAMD software comes with instructions and examples that are complementary to the descriptions presented in this work. Main components of CAMD build on open-source materials science and machine-learning software such as pymatgen,^79^ qmpy,^15^ matminer,^20^ scikit-learn,^80^ gpflow^81^ and tensorflow.^82^ Stability calculations are carried out using a modified (parallelized) implementation of the linear-programming approach in qmpy,^15^ available in the CAMD library. Structure visuals in Fig. 8 are generated using VESTA.^83^ Datasets used or generated in this work are accessible at http://data.matr.io/3. The NN models use a 84 × 50 layer configuration and the default arguments of MLPRegressor class in scikit-learn, with a rectified linear unit activation function, L2 regularization parameter of 0.0001, a learning rate of 0.001 and the *adam* optimizer. The mean absolute error of the models are computed by 3-fold cross validation during each campaign iteration over the seed data to keep track of satisfactory model training and accuracy.

### 5.2 Active campaign settings

In active DFT-based campaigns, agents were allowed to request up to 10 DFT calculations in each iteration. Each campaign is allowed to run for at least 5 iterations regardless of its performance, after which, a campaign would be automatically terminated (i) if the agent could not identify new materials meeting the stability goal within any of the three most recent iterations or (ii) if the campaign reaches 25% consumption of its candidate space or (iii) if the campaign reaches 22 iterations. If an agent cannot suggest at least one structure to acquire with DFT, it terminates the campaign regardless of the campaign step count. Hence, every campaign was allowed effectively a maximum budget of 220 DFT calculations. The stability goal was set as maximum 0.2 eV per atom above the evolving convex-hull. Agents were allowed to choose structures that have up to 20 atoms, and each DFT calculation was allowed a wall-time of 8 hours on 16 CPUs on an AWS EC2 instance. Calculations lingering beyond that point are terminated and agents move to the next stage. While this step is not optimal, and more intelligent stopping criteria for the experiments are planned as future work, the current setting is geared towards the ability to search rapidly and preservation of resources, as relaxations that linger longer often correspond to prototype-derived structures that might be much harder to optimize. Post-campaign unique structure matching tests to determine uniqueness and phase diagram generations are carried out using pymatgen.^79^ Certain agents are allowed to attempt to diversify their requests to minimize the regret of acquiring too many similar candidates using a computationally cheap risk-aversion algorithm. This algorithm measures similarity as Euclidean-distance in a standardized feature space, and finds a diverse subset of the stability-ordered list of candidates meeting the hull-distance threshold. The algorithm eliminates points listed higher on the list (*i.e.* further away from the hull) that are too similar to those lower on this list (*i.e.* closer to the hull), based on a similarity distance threshold that self-adjusts by attempting to iteratively find the smallest such subset larger than the allowed acquisition batch size. The algorithm has no hyperparameters and reduces to the ranked stability list if there are not enough candidates. In a test campaign with a synthetic scenario where we perturbed structure features of materials in the Si–O system to overpopulate the pool with similar candidates, the agent incorporating this algorithm acquired more unique structures.

### 5.3 Generation of search domains for stable material discovery

During formula generation, charge balance was enforced based on the allowed valence state of elements available in pymatgen, if one or more of the elements O, Cl, F, S, N, Br, or I are present in the target chemical system. Formula generation follows a certain set of rules for stoichiometric compound formation. For example, for a system A–B, coefficients *x* and *y* in formulas A_*x*_B_*y*_ are allowed to take integer values from the set {1, 2, …, *g*_max_}. For systems where no charge balance was enforced, the maximum integer, *g*_max_ is set as 4 (inclusive) for both binaries and ternaries. For charge balanced systems, larger values of *g*_max_ are allowed, and *g*_max_ is determined explicitly by incrementing the default maximum above until at least the 20 candidates are generated, upto a hard limit of 7 (inclusive). It should be noted all these parameters can be adjusted by the user, and these are merely the settings we picked for the purposes of the present study.

Structure candidates at each composition are generated with a crystallographic prototype enumeration scheme, as implemented in protosearch^84,85^ using the space group and Wyckoff positions to identify symmetrically unique structures. We have limited the search to experimentally observed crystal structure prototypes, by parsing the ICSD^67^ entries present in the OQMD database,^14,15^ which has a large structural diversity. This consists of approximately 32 000 structures which can be classified into 8050 unique crystal prototypes based on our scheme, including 131 unary (*N* = 1), 1070 binary (*N* = 2), 3196 ternary (*N* = 3), 1970 quaternary (*N* = 4), 1013 quinary (*N* = 5), 542 sexinary (*N* = 6), 104 septenary (*N* = 7), and a few higher-order structures (where N is the number of elemental components). For each selected prototype, a structure (or several structures depending on the symmetry of available sites) with the desired composition is constructed by elemental substitution. Subsequently, lattice constants are approximated by rescaling the unit cell, while avoiding atomic overlap, assuming a hard-sphere radius for atoms as 90% of the elements' covalent radii. Anisotropic scaling is applied to relevant crystal systems, while internal coordinates and angles are kept fixed.

Featurization of all crystal structures (including the OQMD entries used in agent simulations and as seed data, and structures generated for active DFT campaigns as described above) throughout this work was performed using the composition and structure derived (Voronoi-based) material descriptors introduced by Ward *et al.*^66^ producing a vector of 273 features for each material as implemented in Matminer.^20^

### 5.4 Density functional theory calculations

The DFT workflow consists of a structure optimization followed by a static calculation of the final, optimized structure using the Vienna *ab initio* simulation package (VASP).^86,87^ The DFT parameters are generated using OQMD's qmpy interface, which renders OQMD-compatible computational data from the seed data derived from the OQMD.^15^ Note that the actual DFT calculation is done within a docker container on AWS batch, and the Experiment API within CAMD submits, monitors, and fetches the resultant data from the batch job in order to provide formation energy–structure pairs back to the seed dataset.

## Author contributions

JM and MA wrote the python package for agent based computational discovery. KW, RF and TB developed the structure generation methods. JM, JH and MA conceived the project, with input from TB. MA and JM wrote the manuscript, with input from all authors.

## Conflicts of interest

M. A. has a related U.S. patent application. The remaining authors declare no conflict of interest.
